# Supplementary figures and images for: Impact of delayed type hypersensitivity arthritis on development of heart failure by aortic constriction in mice
Source: PLoS One. 2022 Jan 25;17(1):e0262821. doi: 10.1371/journal.pone.0262821 (PMC8789180; doi:10.1371/journal.pone.0262821)

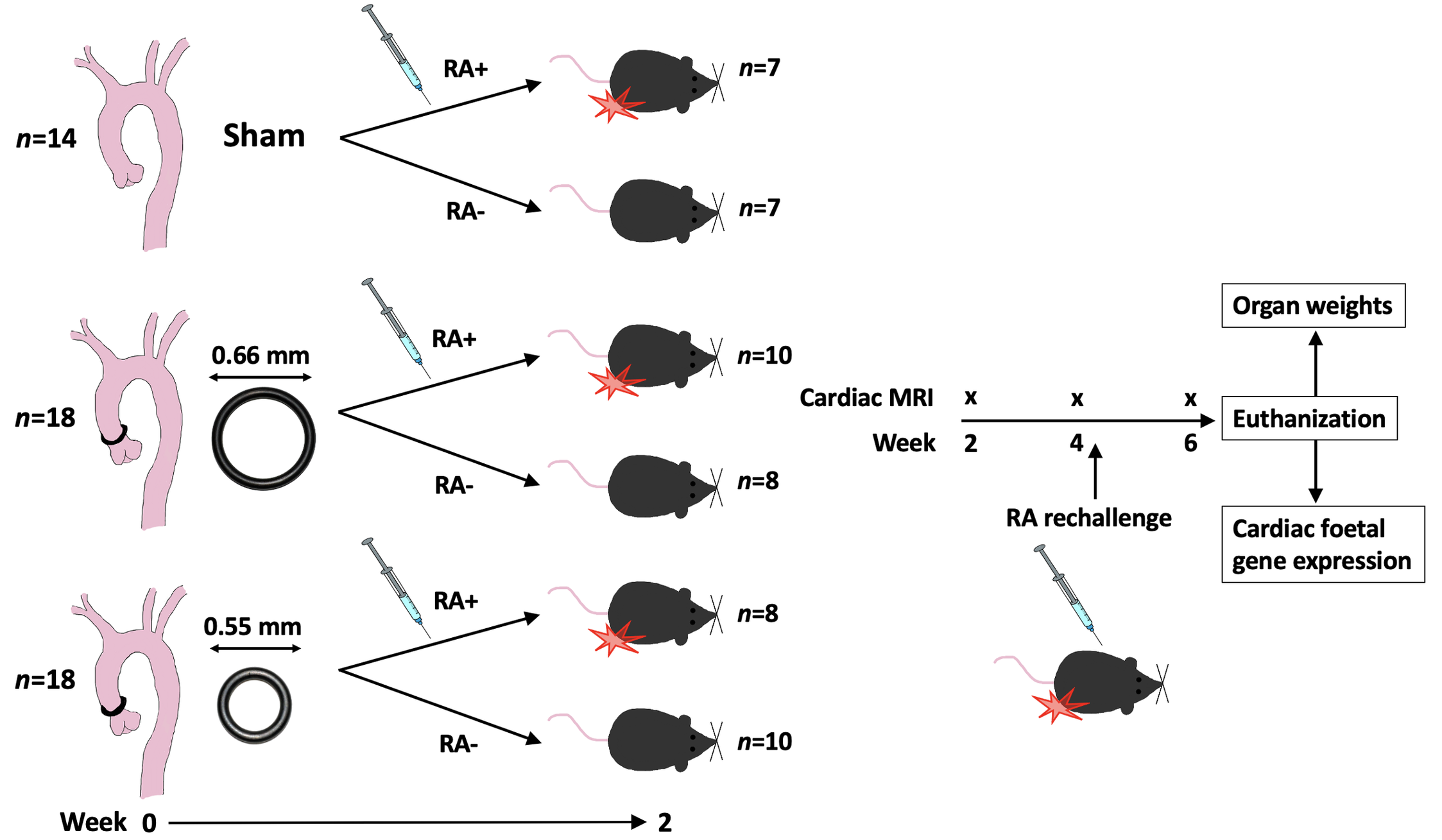

Supplement: S1 Fig — Schematic timeline of the experimental procedures. (TIF) [file pone.0262821.s001.tif]

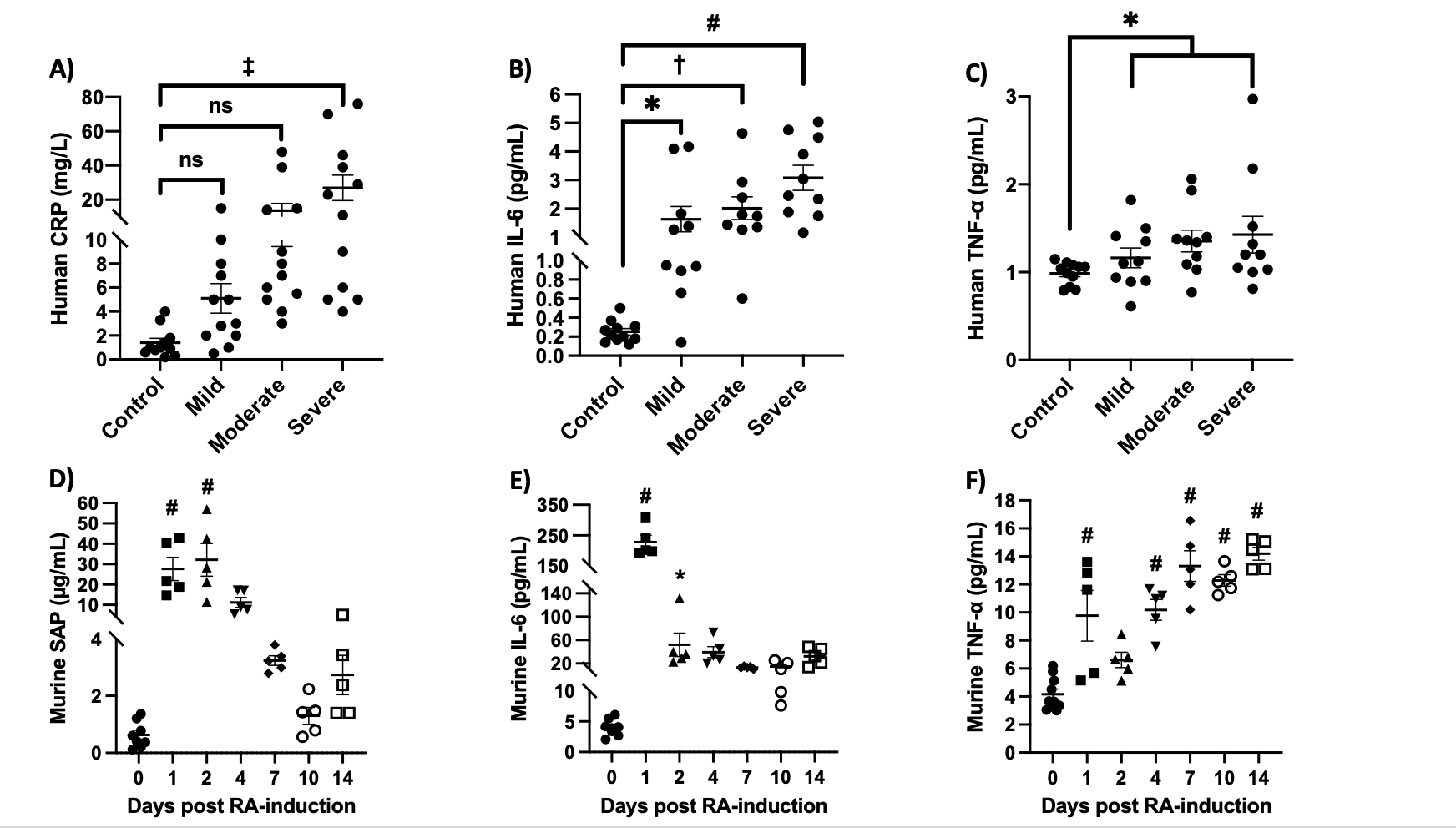

Supplement: S2 Fig — Panel A-C: Serum levels of CRP (panel A), IL-6 (panel B) and TNF-α (panel C) from RA patients. Patients were chosen according to the DAS to represent three disease-states of severity (mild, moderate and severe). Each group consisted of 12 patients and age- and sex-matched healthy volunteers (control). CRP was analysed individually, and included 12 individuals in each group. One outlier was excluded in the healthy volunteer group. Due to space limiting reasons (limited wells in the 96-well) in the analysis of IL-6 and TNF-α, 10 patients were randomized for analysis in each disease group. Of these we excluded one outlier of IL-6 in the moderate group, one outlier of TNF-α in the control group, and one outlier of CRP in the control group. In addition, we excluded one IL-6 value in the control group, even though it did not meet our exclusion criterion, due to that the same individual had outlying both CRP and TNF-α values. Data is displayed as absolute values and presented as scatter plots with mean ± SEM. Statistical significance was calculated using one-way ANOVA with Bonferroni’s multiple comparisons test for CRP and IL-6, and unpaired two-tailed t-test of control vs intervention (mild, moderate and severe combined) for TNF-α. Panel D-F: Temporal profiles of serum levels of murine SAP (panel D), IL-6 (panel E) and TNF-α (panel F) in DTHA mice. Absolute values are shown. n = 10 for control group, n = 5 for other time points. Of these, two outliers for both SAP and IL-6 (representing the same two mice) were excluded in the control groups. Even though it did not meet our exclusion criterion, we chose to also exclude the corresponding TNF-α values from these two mice. Data is shown as scatter plots with mean ± SEM. Statistical significance was calculated using one-way ANOVA with Bonferroni’s multiple comparisons test, relative to control. ns = non-significant, *P < 0.05, †P < 0.01; ‡P < 0.001, #P < 0.0001. DTHA, delayed type hypersensitivity arthritis; CRP, C-reactive pr [file pone.0262821.s002.tif]

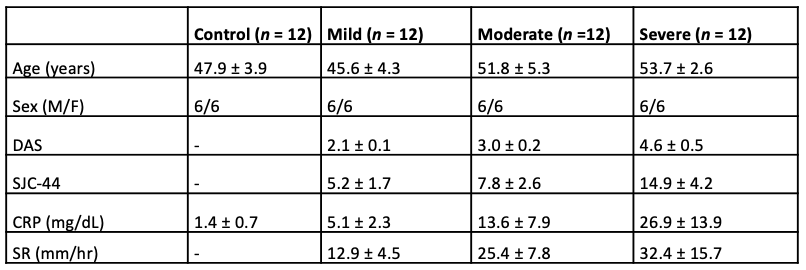

Supplement: S1 Table — Baseline characteristics of patients and healthy controls in the supplemental study. Data is presented as mean values with 95% confidence interval. DAS, Disease Activity Score; SJC-44, swollen joint counts among 44 joints; CRP, C-reactive protein; SR, sedimentation rate. (TIF) [file pone.0262821.s003.tif]
